# Preoperative Inflammatory Markers in Liver Resection for Colorectal Liver Metastases: A National Registry-Based Study

**DOI:** 10.1007/s00268-023-07035-z

**Published:** 2023-05-04

**Authors:** Mushegh A. Sahakyan, Kristoffer Watten Brudvik, Jon-Helge Angelsen, Rachel G. Dille-Amdam, Oddvar M. Sandvik, Bjørn Edwin, Linn S. Nymo, Kristoffer Lassen

**Affiliations:** 1 The Intervention Center, Oslo University Hospital, Rikshospitalet, Oslo, Norway; 2 Department of Research & Development, Division of Emergencies and Critical Care, Oslo University Hospital, Oslo, Norway; 3 Department of Surgery N1, Yerevan State Medical University after M. Heratsi, Yerevan, Armenia; 4 Department of HPB Surgery, Oslo University Hospital, Rikshospitalet, Oslo Norway; 5 Department of Acute and Digestive Surgery, Haukeland University Hospital, Bergen, Norway; 6 Department of Gastrointestinal Surgery, St. Olavs Hospital, Trondheim University Hospital, Trondheim, Norway; 7 Department of Gastrointestinal Surgery, Stavanger University Hospital, Stavanger, Norway; 8 Institute of Clinical Medicine, Medical Faculty, University of Oslo, Oslo, Norway; 9 Department of Gastrointestinal Surgery, University Hospital of North Norway, Tromsø, Norway; 10 Institute of Clinical Medicine, UiT, the Arctic University of Norway, Tromsø, Norway

## Abstract

**Background:**

Preoperative inflammatory markers were shown to be associated with prognosis following surgery for hepato-pancreato-biliary cancer. Yet little evidence exists about their role in patients with colorectal liver metastases (CRLM). This study aimed to examine the association between selected preoperative inflammatory markers and outcomes of liver resection for CRLM.

**Methods:**

Data from the Norwegian National Registry for Gastrointestinal Surgery (NORGAST) was used to capture all liver resections performed in Norway within the study period (November 2015–April 2021). Preoperative inflammatory markers were Glasgow prognostic score (GPS), modified Glasgow prognostic score (mGPS) and C-reactive protein to albumin ratio (CAR). The impact of these on postoperative outcomes, as well as on survival were studied.

**Results:**

Liver resections for CRLM were performed in 1442 patients. Preoperative GPS ≥ 1 and mGPS ≥ 1 were present in 170 (11.8%) and 147 (10.2%) patients, respectively. Both were associated with severe complications but became non-significant in the multivariable model. GPS, mGPS, CAR were significant predictors for overall survival in the univariable analysis, but only CAR remained such in the multivariable model. When stratified by the type of surgical approach, CAR was a significant predictor for survival after open but not laparoscopic liver resections.

**Conclusions:**

GPS, mGPS and CAR have no impact on severe complications after liver resection for CRLM. CAR outperforms GPS and mGPS in predicting overall survival in these patients, especially following open resections. The prognostic significance of CAR in CRLM should be tested against other clinical and pathology parameters relevant for prognosis.

**Supplementary Information:**

The online version contains supplementary material available at 10.1007/s00268-023-07035-z.

## Introduction

Several studies have examined the relationship between preoperative inflammatory markers and prognosis (recurrence, survival) in patients operated for hepato-pancreato-biliary cancer [1–4]. As for patients with colorectal liver metastases (CRLM), there is a limited number of such studies most of which suffer from a relatively small sample size [5–12]. Furthermore, these reports are predominantly based on single-center experiences, which somewhat limits the generalizability of their findings.

Minimally invasive liver resections are increasingly replacing open procedures in the management of CRLM due to less postoperative morbidity, faster recovery and comparable oncologic outcomes [13–16]. At the same time, minimally invasive approach has been shown to be associated with less postoperative inflammatory response compared with open surgery, presumably due to less intraoperative trauma [17]. Thus, the role of preoperative inflammatory markers in CRLM should be considered through the prism of the surgical approach used.

The goal of this study was to examine the association between selected preoperative inflammatory markers and outcomes of liver resection for CRLM using data from a national registry. We also analyzed how the predictive markers performed in subgroups stratified by surgical approach and the extent of liver resection.

## Material and Methods

### Study Design

In this nationwide cohort study, prospectively collected data from the Norwegian National Registry for Gastrointestinal Surgery (NORGAST) was used. Specific information about NORGAST and centralization of the health care system in Norway has been provided elsewhere [18, 19]. This also applies to details about data collection, procedure coding and inclusion/exclusion criteria for NORGAST [18, 20, 21].

**Fig. 1:**
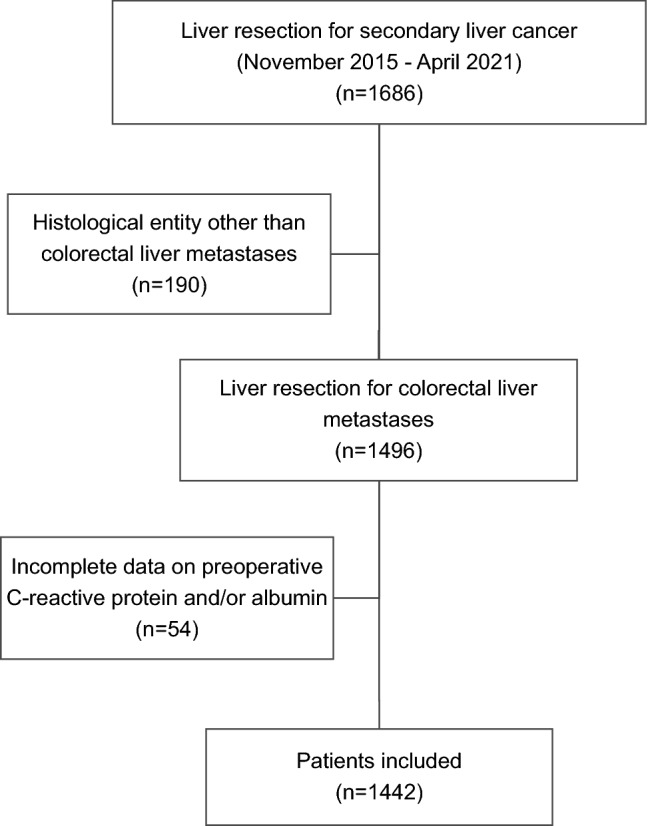
Study flow-chart

Patients with CRLM who had undergone liver resection within the study period (November 2015–April 2021) were included. The associations between preoperative inflammatory markers and postoperative outcomes, as well as survival were studied. Inflammatory markers included Glasgow prognostic score, modified Glasgow prognostic score and C-reactive protein to albumin ratio (GPS, mGPS and CAR, respectively). The last follow-up date was May 31st, 2021. Patients with incomplete information on preoperative C-reactive protein and/or albumin were excluded from the analysis as were those diagnosed with liver lesions of any histological entity other than CRLM (Fig. 1).

The manuscript was written and completed in accordance with the Strengthening the Reporting of Observational Studies in Epidemiology (STROBE) statement [22]. All patients included in NORGAST have given written informed consent for storing their data in the registry. Also, NORGAST holds a data storage license from the Norwegian Data Authority. Finally, this study was approved by the Regional Ethics Committee (2021/268695).

### Definitions

GPS, mGPS and CAR were estimated based upon serum C-reactive protein and albumin levels registered at the last preoperative work-up. GPS 0 denoted normal serum C-reactive protein and albumin levels, while mGPS 0 was used for normal serum C-reactive protein and any albumin levels. GPS and mGPS scored 1 in case of elevated serum C-reactive protein (>10 mg/L) and normal albumin levels. Also, GPS 1 was used for cases with normal C-reactive protein level and hypoalbuminemia (< 35 g/L). Finally, both GPS and mGPS scored 2 if elevated serum C-reactive protein level together with hypoalbuminemia were present. Given the relatively small number of patients with preoperative GPS 2 and mGPS 2, these were studied together with those having GPS 1 and mGPS 1, respectively. This allowed for balancing study groups and reducing the risk for type II error. CAR was analyzed as a continuous variable.

Liver resections were performed either open or laparoscopically. Laparoscopic liver resection was defined as a procedure performed through minimally-invasive approach where laparotomy incisions were done only for either trocar insertion or specimen extraction. Conversion to open surgery was defined as laparotomy at any time during surgery, not specifically related to the extraction of the specimen or trocar insertion. Minor and major hepatectomy included resection of <3 and ≥ 3consecutive liver segments, respectively. Postoperative complications were defined and graded based on the modified Accordion system [23]. Grade ≥ III complications were defined as severe. Postoperative mortality was defined as death within 90 days after surgery. Overall survival was defined as the time between the date of surgery and the date of death from any cause or the date of censoring.

### Statistics

Parameters are presented in the form of continuous or categorical data. Normally distributed continuous data are shown as means (standard deviation), while non-normally distributed (skewed) continuous data are shown as medians (range). Student’s *t*-test and Mann–Whitney *U* test are used for normally and non-normally distributed continuous data, respectively. Categorical data are shown in frequencies (percentages) and analyzed by using the Chi-square and Fisher’s exact tests. A two-tailed *p*-value < 0.05 is considered statistically significant. Parameters significant in the univariable analysis are included in the multivariable binary logistic regression model with backward selection.

The impact of inflammatory markers on survival is examined by using the log-rank test and univariable Cox regression analysis. Parameters significant at *p* < 0.05 in the univariable analyses are entered into multivariable model to identify independent prognostic factors.

## Results

A total number of 1442 patients underwent liver resection for CRLM at five university centers including 311 (21.6%) major hepatectomies. Preoperative GPS was graded as 0, 1 and 2 in 1272 (88.2%), 149 (10.3%) and 21 (1.5%) patients, respectively, while preoperative mGPS was 0, 1 and 2 in 1295 (89.8%), 126 (8.7%) and 21 (1.5%) patients, respectively. Laparoscopic procedures were performed in 720 (49.9%) patients. Severe complications occurred in 255 (17.7%) including 47 (3.3%) reoperations. Ninety-day mortality was observed in 13 (0.9%) cases.

### Inflammatory Markers and Perioperative Results

There were statistically significant differences between the patients with GPS 0 and ≥1 in terms of preoperative weight loss, ECOG score, ASA score, CAR, as well as proportions of laparoscopic and major liver resections (Table 1). Differences in these parameters were also observed in patients with mGPS 0 and mGPS ≥ 1, except weight loss which was similar between the groups. The use of neoadjuvant chemotherapy was more common among the patients with mGPS ≥ 1 compared to those with mGPS 0 (52.4 vs 43.5%, *p* = 0.04).

**Table 1:** Demographics, clinical characteristics, and perioperative data in patients undergoing liver resection for metastases stratified by Glasgow and modified Glasgow prognostic scores

Parameters	Total cohort	GPS^a^ 0	GPS^a^ ≥ 1	*p-*value	mGPS^b^ 0	mGPS^b^ ≥ 1	*p*-value
	*n* = 1442	*n* = 1272	*n* = 170		*n* = 1295	*n* = 147	
Age, years, mean (SD)^c^	65.8 (10.7)	65.7 (10.7)	66.2 (10.8)	0.54	65.8 (10.7)	65.5 (11.1)	0.79
Male sex, *n* (%)^c^	908 (63.2%)	792 (62.5%)	116 (68.2%)	0.15	805 (62.4%)	103 (70.1%)	0.07
BMI, kg/m^2^, mean (SD)	25.9 (4.4)	25.9 (4.4)	26.1 (4.4)	0.67	25.9 (4.5)	26.1 (4.3)	0.51
Weight loss, %, median (range)	2.9 (0–43.4)	2.6 (0–43.4)	5 (0–30.9)	0.003	2.7 (0–43.4)	4.7 (0–27.1)	0.085
Diabetes, *n* (%)	126 (8.7%)	110 (8.6%)	16 (9.4%)	0.74	113 (8.7%)	13 (8.8%)	0.96
Severe lung disease, *n* (%)	12 (0.8%)	9 (0.7%)	3 (1.8%)	0.16	9 (0.7%)	3 (2%)	0.2
Severe cardiac disease, *n* (%)	23 (1.6%)	19 (1.5%)	4 (2.4%)	0.34	20 (1.5%)	3 (2%)	0.72
Neoadjuvant chemotherapy, *n* (%)	640 (44.4%)	554 (43.6%)	86 (50.6%)	0.083	563 (43.5%)	77 (52.4%)	0.04
ECOG score, *n* (%)^c^				0.001			0.001
0	1033 (73.3%)	949 (76.3%)	84 (50.3%)		959 (75.8%)	74 (51.1%)	
1	319 (22.6%)	252 (20.3%)	67 (40.1%)		263 (20.8%)	56 (38.6%)	
≥2	58 (4.1%)	42 (3.4%)	16 (9.6%)		43 (3.4%)	15 (10.3%)	
ASA score ≥ III, *n* (%)	659 (45.7%)	553 (43.5%)	106 (62.4%)	0.001	570 (44%)	89 (60.5%)	0.001
CAR, median (range)^d^	0.073 (0.02–8.15)	0.05 (0.02–0.28)	0.5 (0.03–6.96)	0.001	0.05 (0.02–0.33)	0.56 (0.24–6.97)	0.001
Major resection, *n* (%)	311 (21.6%)	253 (19.9%)	58 (34.1%)	0.001	256 (19.8%)	55 (37.4%)	0.001
Laparoscopic resection, *n* (%)	720 (49.9%)	654 (51.4%)	66 (38.8%)	0.002	665 (51.4%)	55 (37.4%)	0.001
Conversion, *n* (%)	41 (5.7%)	40 (6.1%)	1 (1.5%)	0.16	40 (6%)	1 (1.8%)	0.36

^a^Glasgow prognostic score; ^b^modified Glasgow prognostic score; ^c^incomplete data; ^d^CRP-to-albumin ratio

Patients with severe complications had more weight loss, greater proportion of severe lung diseases and more frequent use of neoadjuvant chemotherapy (Table 2). Both GPS ≥ 1 and mGPS ≥ 1 were associated with severe complications. So were weight loss, presence of severe lung disease, neoadjuvant chemotherapy, and performing major liver resection. The latter turned out to be the only predictor for severe complications in the multivariable analysis.

**Table 2:** Uni- and multivariable analyses of factors associated with severe complications after liver resection for colorectal liver metastases

Parameters	Severe complications		*p*-value	Multivariable model^a^	*p*-value
	Yes (*n* = 255)	No (*n* = 1187)		Odds ratio (95% CI)	
Age, years, mean (SD)^b^	66.2 (11.1)	65.7 (10.7)	0.47		
Male sex, *n* (%)^b^	174 (68.2%)	734 (62.1%)	0.065		
BMI, kg/m^2^, mean (SD)	25.8 (4.4)	25.9 (4.4)	0.55		
Weight loss, %, median (range)	4.6 (0–30.9)	2.4 (0–43.4)	0.003	1.02 (0.99–1.05)	0.087
Diabetes, *n* (%)	22 (8.6%)	104 (8.8%)	0.95		
Severe lung disease, *n* (%)	6 (2.4%)	6 (0.5%)	0.01	–	–
Severe cardiac disease, *n* (%)	7 (2.7%)	16 (1.3%)	0.16		
Neoadjuvant chemotherapy, *n* (%)	137 (53.7%)	503 (42.4%)	0.001	–	–
ECOG score, *n* (%)^b^			0.11		
0	175 (70.3%)	858 (73.9%)			
1	58 (23.3%)	261 (22.5%)			
≥ 2	16 (6.4%)	42 (3.6%)			
ASA score ≥ III, *n* (%)	127 (49.8%)	532 (44.8%)	0.15		
GPS^c^, *n* (%)			0.033		
0	215 (84.3%)	1057 (89%)		Reference	
≥1	40 (15.7%)	130 (11%)		–	–
mGPS^d^, *n* (%)			0.012		
0	218 (85.5%)	1077 (90.7%)		Reference	
≥1	37 (14.5%)	110 (9.3%)		–	–
CAR^e^, median (range)	0.07 (0.02–6.81)	0.07 (0.02–6.97)	0.31		
Major resection, *n* (%)	94 (36.9%)	217 (18.3%)	0.001	3.39 (2.32–4.94)	0.001
Conversion, *n* (%)	6 (7.2%)	35 (5.5%)	0.46		

^a^Backward selection (parameters significant at p-value < 0.05 in the univariable analysis included); ^b^Incomplete data; ^c^Glasgow prognostic score; ^d^modified Glasgow prognostic score; ^e^CRP-to-albumin ratio

Analysis of specific types of severe complications demonstrated that GPS and mGPS were associated with single-organ failure after liver resection (suppl. Table 1). However, these associations were not statistically significant in the multivariable model (suppl. table 2). Subgroup analyses in patients undergoing minor/major liver resection and open/laparoscopic surgery did not reveal any statistically significant association between the inflammatory markers and severe complications (suppl. Tables 3, 4, 5, 6).

### Inflammatory Markers and Survival

**Fig. 2:**
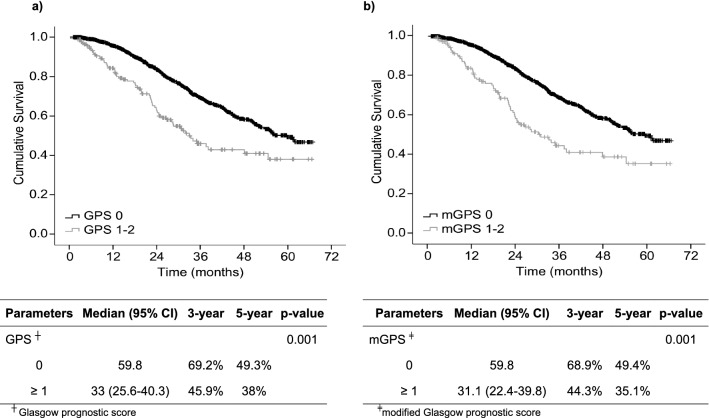
Inflammation-based prognostic scores and overall survival in patients undergoing liver resection for colorectal liver metastases—Glasgow prognostic score (**a**) and modified Glasgow prognostic score (**b**)

Median follow-up was 25 (1–67) months. Three- and 5-year survival were 66.4% and 47.9%, respectively. Both GPS 0 and mGPS 0 resulted in significantly longer survival compared with GPS ≥ 1 and mGPS ≥ 1, respectively (Fig. 2). Parameters such as age, ECOG score, ASA score, CAR and severe complications were also associated with survival in the univariable analysis (Table 3). In the multivariable model, age, ECOG ≥ 2, ASA, CAR and severe complications were the only significant prognostic factors.

**Table 3:** Uni- and multivariable Cox regression analyses of factors associated with overall survival after liver resection for colorectal liver metastases

Parameters	Univariable analysis		Multivariable analysis	
	Hazard ratio (95% CI)	*p*-value	Hazard ratio (95% CI)	*p*-value
Age	1.02 (1.01–1.03)	0.001	1.01 (1.002–1.023)	0.017
Sex (male)	1.11 (0.91–1.37)	0.3		
BMI	0.98 (0.96–1.01)	0.13		
Weight loss	1.01 (0.98–1.03)	0.55		
Diabetes	1.15 (0.82–1.61)	0.41		
Severe lung disease	1.52 (0.68–3.41)	0.31		
Severe cardiac disease	1.08 (0.59–1.98)	0.79		
ECOG score (vs 0)				
1	1.43 (1.14–1.80)	0.002	1.22 (0.96–1.54)	0.09
≥2	2.11 (1.39–3.18)	0.001	1.57 (1.02–2.4)	0.04
ASA score ≥ III, *n* (%)	1.56 (1.29–1.91)	0.001	1.29 (1.04–1.6)	0.019
GPS ≥ 1 (vs 0) ^a^	2.02 (1.56–2.62)	0.001	1.16 (0.55–2.47)	0.7
mGPS ≥ 1 (vs 0) ^b^	2.17 (1.66–2.84)	0.001	1.34 (0.6–2.98)	0.47
CAR	1.62 (1.42–1.85)	0.001	1.32 (1.09–1.59)	0.004
Major resection	1.13 (0.9–1.43)	0.29		
Severe complication	1.58 (1.25–1.98)	0.001	1.53 (1.21–1.94)	0.001

^a^Glasgow prognostic score; ^b^modified Glasgow prognostic score; ^c^CRP-to-albumin ratio

Subgroup analyses were performed in patients undergoing laparoscopic and open resection for CRLM (Table 4). In the multivariable analysis, CAR was the only prognostic factor following open resections (1.41 (1.14–1.73), *p* = 0.001). For laparoscopic resections, ECOG ≥ 2 and severe complications were the only significant prognostic factors in the multivariable model. In patients undergoing minor hepatectomy, factors such as age, ECOG ≥ 2, ASA score, CAR and severe complications were significant for prognosis (suppl. table 4). As for major hepatectomy, the impact of CAR was marginal (1.27 (0.99–1.63), *p* = 0.055), while other parameters were not significant in the multivariable analysis.

**Table 4:** Uni- and multivariable Cox regression analyses of factors associated with overall survival after open and laparoscopic liver resection for colorectal liver metastases

Parameters	Open				Laparoscopic			
	Univariable analysis		Multivariable analysis		Univariable analysis		Multivariable analysis	
	HR (95% CI)	*p*-value	HR (95% CI)	*p*-value	HR (95% CI)	*p*-value	HR (95% CI)	*p*-value
Age	1.02 (1.01–1.03)	0.007	1.01 (0.99–1.03)	0.07	1.02 (1.01–1.04)	0.004	1.01 (0.99–1.03)	0.1
Sex (male)	1.09 (0.84–1.43)	0.49			1.13 (0.82–1.56)	0.46		
BMI	0.98 (0.95–1.01)	0.12			0.98 (0.95–1.02)	0.42		
Weight loss	0.99 (0.96–1.03)	0.73			1.02 (0.99–1.06)	0.21		
Diabetes	1.05 (0.68–1.63)	0.82			1.29 (0.77–2.15)	0.34		
Severe lung disease	1.18 (0.44–3.18)	0.74			2.5 (0.62–10.1)	0.2		
Severe cardiac disease	0.94 (0.49–1.78)	0.85			1.36 (0.19–9.75)	0.76		
ECOG score (vs 0)								
1	1.41 (1.06–1.88)	0.02	1.27 (0.94–1.7)	0.12	1.41 (0.98–2.05)	0.07	1.1 (0.74–1.64)	0.63
≥ 2	1.71 (0.99–2.96)	0.049	1.33 (0.75–2.34)	0.33	2.71 (1.45–5.06)	0.002	1.98 (1.03–3.79)	0.04
ASA score ≥ III, *n* (%)	1.44 (1.11–1.86)	0.006	1.25 (0.95–1.64)	0.11	1.69 (1.25–2.31)	0.001	1.29 (0.91–1.84)	0.15
GPS ≥ 1 (vs 0)	1.89 (1.38–2.6)	0.001	1.48 (0.65–3.38)	0.35	2.11 (1.34–3.31)	0.001	0.51 (0.07–3.67)	0.5
mGPS ≥ 1 (vs 0)	1.89 (1.36–2.64)	0.001	0.88 (0.36–2.11)	0.77	2.59 (1.64–4.11)	0.001	3.54 (0.45–27.9)	0.23
CAR	1.58 (1.35–1.85)	0.001	1.41 (1.14–1.73)	0.001	2.58 (1.68–3.97)	0.001	1.51 (0.77–2.96)	0.24
Major resection	1.08 (0.82–1.41)	0.59			0.96 (0.55–1.65)	0.87		
Severe complications	1.36 (1.02–1.81)	0.03	1.32 (0.98–1.78)	0.06	1.93 (1.31–2.85)	0.001	2.01 (1.35–2.99)	0.001

## Discussion

This study suggests that preoperative CAR outperforms GPS and mGPS when considering their impact on overall survival after liver resection for CRLM. To the best of our knowledge, this is the largest study assessing the prognostic role of preoperative inflammatory markers in patients operated for CRLM. Furthermore, this is the first report examining the performances of all C-reactive protein and albumin-based inflammatory markers (GPS, mGPS and CAR) in these patients. Unlike previously published reports our study includes a complete national dataset allowing for a full coverage of CRLM resections performed on a nationwide basis within the study period. Our findings do not agree with those reported by Solaini and co-workers, who found that preoperative GPS was more sensitive than CAR in predicting overall survival [8]. Nevertheless, our results are in line with those from Haruki et al. suggesting better prognostic ability for CAR compared with mGPS [6].

Our findings indicate that the prognostic role of preoperative CAR is relevant for open, but not for laparoscopic liver resections. As mentioned above, reduced systemic inflammation was observed following laparoscopic liver resection for CRLM when compared with its open counterpart [17]. Building upon these and our findings, one may assume that while the negative impact of preoperative inflammation is further aggravated by open surgery, laparoscopy may alleviate these effects, thereby providing benefits in patients with increased preoperative CAR. We also found that severe complications led to worse prognosis in patients undergoing laparoscopic surgery, but not in those undergoing open surgery. Putting this in the context of surgical technique and postoperative inflammation, one can speculate that severe complications arising after laparoscopic resections nullify or significantly diminish the inflammation-related benefits of laparoscopy, while such changes are less pronounced for open surgery. These hypotheses require further investigation focused specifically on perioperative changes in inflammatory markers, their relationship with surgical technique, complications, and prognosis.

This study also assessed the associations between the preoperative inflammatory markers and perioperative outcomes. GPS ≥ 1 but not mGPS ≥ 1 was associated with preoperative weight loss, while none of them was linked to body mass index before surgery. Preoperative weight loss was a significant predictor for severe complications in the univariable analysis although not statistically significant in the final model. These findings require further scrutiny as weight loss may indicate patient frailty and possible increased risk for postoperative complications. Both GPS ≥ 1 and mGPS ≥ 1 were associated with higher ECOG and ASA scores, as well as with performing open surgery and major hepatectomy. The last two might be surrogate markers of disease spread or greater tumor size. Unfortunately, NORGAST does not hold data for TNM-stage, so this assumption cannot be tested. At the same time, none of the preoperative inflammatory markers was associated with severe complications following surgery. Subgroup analyses in patients undergoing minor and major hepatectomy also did not reveal any association between the preoperative inflammatory markers and postoperative complications.

There are several important limitations with this study. Primarily, although data collection was conducted prospectively in the registry, the protocol for analysis was constructed after the inclusion period. Secondly, as NORGAST is designed for all types of gastro-intestinal and hepato-pancreato-biliary resections, several CRLM-specific pathology and clinical parameters were not registered. Also, molecular prognostic factors in CRLM such as RAS status, bRAF and MSI have not been a part of this registry. That somewhat limits the reliability and interpretation of prognostic factors determined in the multivariable analysis. It would be desirable to test the prognostic significance of CAR by analyzing it together with all the clinical, pathology and molecular parameters relevant for CRLM. Third, data on recurrence, its site and recurrence-free survival was not registered, so these parameters could not be studied in the context of preoperative inflammatory markers. Finally, data on some baseline characteristics were incomplete in a negligible proportion of cases (< 1%).

## Conclusions

Preoperative inflammatory markers have no correlation with postoperative severe complications in patients undergoing liver resection for CRLM. Preoperative CAR outperforms GPS and mGPS in predicting overall survival following liver resection for CRLM, especially for patients undergoing open procedures. The prognostic significance of CAR needs to be tested against other clinical, pathology and molecular parameters that are relevant for prognosis.

## Supplementary Information

Below is the link to the electronic supplementary material.

Supplementary file1 (DOCX 35 KB)
