# Supplementary material for: Preoperative Inflammatory Markers in Liver Resection for Colorectal Liver Metastases: A National Registry-Based Study
Source: World J Surg. 2023 May 4;47(9):2213–20. doi: 10.1007/s00268-023-07035-z (PMC10387457; doi:10.1007/s00268-023-07035-z)
Supplement: Supplementary file 1 — Supplementary file1 (DOCX 35 KB) [file 268_2023_7035_MOESM1_ESM.docx]

**Supplementary Table 1. Associations between severe complications after liver resection for colorectal liver metastases and preoperative inflammatory markers (a - Glasgow and modified Glasgow prognostic scores; b – C-reactive protein-to-albumin ratio).**

**a)**

| **Parameters** | **Total** | **GPS^┼^** | | | **mGPS^╪^** | | |
| --- | --- | --- | --- | --- | --- | --- | --- |
|  | **(n=1442)** | **0**  **(n=1272)** | **≥ 1**  **(n=170)** | **p-value** | **0**  **(n=1295)** | **≥ 1**  **(n=147)** | **p-value** |
| Relaparotomy, n (%) | 47 (3.3%) | 37 (2.9%) | 10 (5.9%) | 0.06 | 39 (3%) | 8 (5.4%) | 0.14 |
| Single-organ failure, n (%) | 40 (2.8%) | 30 (2.4%) | 10 (5.9%) | 0.02 | 31 (2.4%) | 9 (6.1%) | 0.016 |
| Multi-organ failure, n (%) | 9 (0.6%) | 7 (0.6%) | 2 (1.2%) | 0.29 | 8 (0.6%) | 1 (0.7%) | 1.0 |

**^┼^**Glasgow prognostic score; **^╪^** modified Glasgow prognostic score.

**b)**

| **Parameters** | **Relaparotomy** | | **Single-organ failure** | | **Multiorgan failure** | |
| --- | --- | --- | --- | --- | --- | --- |
|  | **Y=47** | **N=1395** | **Y=40** | **N=1402** | **Y=9** | **N=1433** |
| CAR**^¶^**, median (range) | 0.07 (0.02-2.31) | 0.07 (0.02-8.15) | 0.09 (0.02-2.31) | 0.07 (0.02-8.15) | 0.06 (0.02-0.63) | 0.07 (0.02-8.15) |

**^¶^** CRP-albumin ratio

**Supplementary Table 2. Uni- and multivariable analyses of risk factors for single-organ failure following liver resection for colorectal liver metastases.**

|  | ***Single-organ failure*** | | | |
| --- | --- | --- | --- | --- |
| **Parameters** | **Univariable analysis** | **p-value** | **Multivariable analysis**¹ | **p-value** |
|  | **Odds ratio (95% CI)** |  | **Odds ratio (95% CI)** |  |
| Age, years | 1.05 (1.01-1.09) | 0.007 | 1.05 (1.01-1.09) | 0.018 |
| Sex (male) | 2.38 (1.09-5.2) | 0.03 | 2.18 (0.97-4.85) | 0.06 |
| BMI, kg/m^2^ | 1.02 (0.95-1.09) | 0.58 |  |  |
| Weight loss, % | 1.03 (0.96-1.09) | 0.43 |  |  |
| Diabetes | 1.89 (0.78-4.58) | 0.16 |  |  |
| Severe lung disease | 19.3 (5.6-67.2) | 0.001 | 15.6 (3.8-64.4) | 0.001 |
| Severe cardiac disease | 5.6 (1.6-19.7) | 0.007 | ___________ | - |
| Neoadjuvant chemotherapy | 1.91 (1.01-3.64) | 0.047 | 2.16 (1.07-4.32) | 0.03 |
| ECOG score (vs ECOG 0) |  |  |  |  |
| 1 | 2.15 (1.06-4.38) | 0.034 | 1.83 (0.89-3.79) | 0.1 |
| ≥ 2 | 6.95 (2.81-17.2) | 0.001 | 5.29 (1.95-14.4) | 0.001 |
| ASA score ≥ III, n (%) | 2.02 (1.06-3.86) | 0.034 | ___________ | - |
| GPS^┼^ ≥ 1 (vs GPS 0) | 2.59 (1.24-5.39) | 0.011 | ___________ | - |
| mGPS^╪^ ≥ 1 (vs mGPS 0) | 2.66 (1.24-5.7) | 0.012 | ___________ | - |
| CAR^*^ | 1.24 (0.85-1.79) | 0.26 |  |  |
| Major resection | 2.24 (1.17-4.31) | 0.015 | 2.22 (1.1-4.5) | 0.026 |
| Conversion | 1.67 (0.21-13.4) | 0.63 |  |  |

¹ Backward selection (parameters significant at p-value < 0.05 in the univariable analysis included); ^┼^ Glasgow prognostic score; ^╪^ modified Glasgow prognostic score; * CRP-to-albumin ratio.

**Supplementary Table 3. Associations between the inflammation-based prognostic systems and severe complications after major and minor liver resection for colorectal liver metastases.**

| **Parameters** | *Major (n=311)* | | | | *Minor (n=1131)* | | | |
| --- | --- | --- | --- | --- | --- | --- | --- | --- |
|  | **Yes** | **No** | | **p-value** | **Yes** | **No** | | **p-value** |
|  | **(n=94)** | | **(n=217)** |  | **(n=161)** | | **(n=970)** |  |
| GPS^┼^, (%) |  | |  | 0.43 |  | |  | 0.25 |
| 0 | 74 (78.7%) | | 179 (82.5%) |  | 141 (87.6%) | | 878 (90.5%) |  |
| ≥ 1 | 20 (21.3%) | | 38 (17.5%) |  | 20 (12.4%) | | 92 (9.5%) |  |
| mGPS^╪^, (%) ^╪^ |  | |  | 0.28 |  | |  | 0.22 |
| 0 | 74 (78.7%) | | 182 (83.9%) |  | 144 (89.4%) | | 895 (92.3%) |  |
| ≥ 1 | 20 (21.3%) | | 35 (16.1%) |  | 17 (10.6%) | | 75 (7.7%) |  |
| CAR*, median (range) | 0.1 (0.02-8.15) | | 0.095 (0.02-6.04) | 0.53 | 0.69 (0.02-1.34) | | 0.71 (0.02-6.97) | 0.89 |

^┼^ Glasgow prognostic score; ^╪^ modified Glasgow prognostic score; * CRP-to-albumin ratio.

**Supplementary Table 4. Associations between the inflammation-based prognostic systems and severe complications after open and laparoscopic liver resection for colorectal liver metastases.**

| **Parameters** | *Open (n=722)* | | | | *Laparoscopic (n=720)* | | | |
| --- | --- | --- | --- | --- | --- | --- | --- | --- |
|  | **Yes** | **No** | | **p-value** | **Yes** | **No** | | **p-value** |
|  | **(n=172)** | | **(n=550)** |  | **(n=83)** | | **(n=637)** |  |
| GPS^┼^, (%) |  | |  | 0.07 |  | |  | 0.87 |
| 0 | 140 (81.4%) | | 478 (86.9%) |  | 75 (90.4%) | | 579 (90.9%) |  |
| ≥ 1 | 32 (18.6%) | | 72 (13.1%) |  | 8 (9.6%) | | 58 (9.1%) |  |
| mGPS^╪^, (%) |  | |  | 0.03 |  | |  | 0.77 |
| 0 | 142 (82.6%) | | 488 (88.7%) |  | 76 (91.6%) | | 589 (92.5%) |  |
| ≥ 1 | 30 (17.4%) | | 62 (11.3%) |  | 7 (8.4%) | | 48 (7.5%) |  |
| CAR*, median (range) | 0.096 (0.02-8.15) | | 0.1 (0.02-6.04) | 0.47 | 0.05 (0.02-1.24) | | 0.06 (0.02-6.97) | 0.51 |

^┼^ Glasgow prognostic score; ^╪^ modified Glasgow prognostic score; * CRP-to-albumin ratio.

**Supplementary Table 5. Uni- and multivariable analyses of factors associated with severe complications after open liver resection for colorectal liver metastases.**

| **Parameters** |  | **Severe complications** | | **p-value** | **Multivariable model** | **p-value** |
| --- | --- | --- | --- | --- | --- | --- |
|  |  | **Yes (n=172)** | **No (n=550)** |  | **Odds ratio (95% CI)** |  |
| Age, years, mean (SD) ^¶^ | | 65.2 (11.1) | 64.8 (10.4) | 0.65 |  |  |
| Sex (male), n (%) ^¶^ | | 115 (61.2%) | 335 (61.2%) | 0.18 |  |  |
| BMI, kg/m^2^, mean (SD) | | 26.2 (4.6) | 26.4 (4.8) | 0.63 |  |  |
| Weight loss, %, median (range) | | 4.7 (0-27.4) | 2.6 (0-43.4) | 0.042 | 1.02 (0.98-1.06) | 0.32 |
| Diabetes, n (%) | | 16 (9.3%) | 48 (8.7%) | 0.82 |  |  |
| Severe lung disease, n (%) | | 4 (2.3%) | 4 (0.7%) | 0.1 |  |  |
| Severe cardiac disease, n (%) | | 5 (2.9%) | 15 (2.7%) | 1.0 |  |  |
| Neoadjuvant chemotherapy, n (%) | | 101 (58.7%) | 270 (49.1%) | 0.027 | 1.12 (0.69-1.83) | 0.65 |
| ECOG score, n (%) ^¶^ | |  |  | 0.29 |  |  |
| 0 | | 116 (68.6%) | 395 (72.9%) |  |  |  |
| 1 | | 42 (24.9%) | 126 (23.2%) |  |  |  |
| ≥ 2 | | 11 (6.5%) | 21 (3.9%) |  |  |  |
| ASA score ≥ III, n (%) | | 88 (51.2%) | 255 (46.4%) | 0.27 |  |  |
| GPS^┼^, n (%) | |  |  | 0.07 |  |  |
| 0 | | 140 (81.4%) | 478 (86.9%) |  |  |  |
| ≥ 1 | | 32 (18.6%) | 72 (13.1%) |  |  |  |
| mGPS^╪^, n (%) | |  |  | 0.03 |  |  |
| 0 | | 142 (82.6%) | 488 (88.7%) |  | reference |  |
| ≥ 1 | | 30 (17.4%) | 62 (11.3%) |  | 1.05 (0.53-2.09) | 0.89 |
| CAR^*^, median (range) | | 0.096 (0.02-8.15) | 0.1 (0.02-6.04) | 0.47 |  |  |
| Major resection, n (%) | | 76 (44.2%) | 169 (30.7%) | 0.001 | 2.28 (1.39-3.73) | 0.001 |

^¶^ Incomplete data; ^┼^ Glasgow prognostic score; ^╪^ modified Glasgow prognostic score; * CRP-to-albumin ratio.

**Supplementary Table 6. Uni- and multivariable Cox regression analyses of prognostic factors after minor and major hepatectomy for colorectal liver metastases.**

| **Parameters** | ***Minor*** | | | | ***Major*** | | | |
| --- | --- | --- | --- | --- | --- | --- | --- | --- |
|  | **Univariable analysis** | | **Multivariable analysis** | | **Univariable analysis** | | **Multivariable analysis** | |
|  | **HR (95% CI)** | **p-value** | **HR (95% CI)** | **p-value** | **HR (95% CI)** | **p-value** | **HR (95% CI)** | **p-value** |
| Age | 1.02 (1.01-1.03) | 0.001 | 1.02 (1.01-1.03) | 0.013 | 1.01 (0.99-1.03) | 0.26 |  |  |
| Sex (male) | 1.06 (0.84-1.33) | 0.65 |  |  | 1.33 (0.87-2.04) | 0.19 |  |  |
| BMI | 0.98 (0.95-1.01) | 0.09 |  |  | 1.01 (0.96-1.05) | 0.96 |  |  |
| Weight loss | 1.01 (0.98-1.03) | 0.75 |  |  | 1.02 (0.97-1.08) | 0.44 |  |  |
| Diabetes | 1.13 (0.76-1.66) | 0.55 |  |  | 1.24 (0.64-2.38) | 0.53 |  |  |
| Severe lung disease | 1.55 (0.69-3.49) | 0.29 |  |  | ___________ | - |  |  |
| Severe cardiac disease | 1.08 (0.55-2.01) | 0.82 |  |  | 1.14 (0.28-4.64) | 0.85 |  |  |
| ECOG score (vs 0) |  |  |  |  |  |  |  |  |
| 1 | 1.39 (1.07-1.82) | 0.013 | 1.19 (0.9-1.57) | 0.22 | 1.53 (0.98-2.42) | 0.064 |  |  |
| ≥ 2 | 1.97 (1.25-3.12) | 0.004 | 1.64 (1.03-2.63) | 0.038 | 2.95 (1.17-7.45) | 0.022 | 1.76 (0.65-4.82) | 0.27 |
| ASA score ≥ III, n (%) | 1.59 (1.27-1.99) | 0.001 | 1.29 (1.01-1.65) | 0.04 | 1.47 (0.98-2.2) | 0.064 |  |  |
| GPS^┼^ ≥ 1 (vs 0) | 1.73 (1.26-2.38) | 0.001 | 1.12 (0.46-2.73) | 0.81 | 2.78 (1.76-4.39) | 0.001 | 1.69 (0.41-6.91) | 0.47 |
| mGPS^╪^ ≥ 1 (vs 0) | 1.86 (1.33-2.60) | 0.001 | 1.04 (0.39-2.80) | 0.94 | 2.91 (1.82-4.66) | 0.001 | 1.2 (0.27-5.28) | 0.81 |
| CAR* | 2.33 (1.64-2.39) | 0.001 | 1.8 (1.04-3.1) | 0.035 | 1.49 (1.25-1.78) | 0.001 | 1.27 (0.99-1.63) | 0.055 |
| Laparoscopic approach | 0.81 (0.65-1.01) | 0.065 |  |  | 0.72 (0.41-1.26) | 0.25 |  |  |
| Severe complications | 1.98 (1.51-2.59) | 0.001 | 2.03 (1.54-2.66) | 0.001 | 0.93 (0.59-1.44) | 0.74 |  |  |

^┼^ Glasgow prognostic score; ^╪^ modified Glasgow prognostic score; * CRP-to-albumin ratio.
